# Combination of measures distinguishes pre-miRNAs from other stem-loops in the genome of the newly sequenced *Anopheles darlingi*

**DOI:** 10.1186/1471-2164-11-529

**Published:** 2010-09-29

**Authors:** Nuno D Mendes, Ana T Freitas, Ana T Vasconcelos, Marie-France Sagot

**Affiliations:** 1 Équipe BAOBAB, Laboratoire de Biométrie et Biologie Évolutive (UMR 5558); CNRS; Univ. Lyon 1, 43 bd du 11 nov 1918, 69622, Villeurbanne Cedex, France; 2 IST/INESC-ID, 9 Rua Alves Redol, 1000-029 Lisbon, Portugal; 3 BAMBOO Team, INRIA Rhone-Alpes, 655 avenue de l'Europe, 38330 Montbonnot Saint-Martin, France; 4 Bioinformatics Laboratory, National Laboratory of Scientific Computation (LNCC), Avenida Getúlio Vargas, 333, Petrópolis, Brazil

## Abstract

**Background:**

Efforts using computational algorithms towards the enumeration of the full set of miRNAs of an organism have been limited by strong reliance on arguments of precursor conservation and feature similarity. However, miRNA precursors may arise anew or be lost across the evolutionary history of a species and a newly sequenced genome may be evolutionarily too distant from other genomes for an adequate comparative analysis. In addition, the learning of intricate classification rules based purely on features shared by miRNA precursors that are currently known may reflect a perpetuating identification bias rather than a sound means to tell true miRNAs from other genomic stem-loops.

**Results:**

We show that there is a strong bias amongst annotated pre-miRNAs towards robust stem-loops in the genomes of *Drosophila melanogaster *and *Anopheles gambiae *and we propose a scoring scheme for precursor candidates which combines four robustness measures. Additionally, we identify several known pre-miRNA homologs in the newly sequenced *Anopheles darlingi *and show that most are found amongst the top-scoring precursor candidates. Furthermore, a comparison of the performance of our approach is made against two single-genome pre-miRNA classification methods.

**Conclusions:**

In this paper we present a strategy to sieve through the vast amount of stem-loops found in metazoan genomes in search of pre-miRNAs, significantly reducing the set of candidates while retaining most known miRNA precursors. This approach makes no use of conservation data and relies solely on properties derived from our knowledge of miRNA biogenesis.

## Background

Animal miRNAs are small molecules with ~22-nt in length playing an important role in the post-transcriptional regulation of gene expression. They originate from the maturation of larger precursor molecules called pre-miRNAs with ~70-nt in length and a typical foldback structure. These stem-loops can be excised from larger primary transcripts often containing several precursors in tandem [1], or from the introns of protein-coding genes (in some restricted cases, as a result of splicing [2]).

The biological function of miRNAs is defined by the genes they target by imperfectly pairing with binding sites located primarily in the 3'UTR region of the target gene [1]. A set of these regulators will usually act in coordination to dampen or abolish the expression of a common target, although a single miRNA may efficiently silence a gene [3].

The identification of the full set of miRNAs of an organism is an important step towards the understanding of complex gene regulatory networks of which miRNA-dependent silencing is a crucial aspect. Since mature miRNAs are too small to exhibit distinguishable features, the computational search for these regulators has focused on the identification of their larger precursors and their characteristic hairpin secondary structure [4].

Despite a growing list of miRNAs, identified either by experimental assays or using current computational tools, the goal of enumerating the full catalogue of miRNAs of any single organism has proven to be difficult, requiring different approaches to identify a decreasing number of novel regulators. A recent thorough experimental study of mammalian miRNAs did find new regulators, but it also showed that several annotated sequences were likely not miRNAs [5]. The difficulties are two-fold. On the one hand, purely experimental detection is limited to miRNAs which are expressed at relatively high levels and in broad cellular types/conditions. Recent deep-sequencing techniques tackle these limitations but require extensive computational analyses [6]. On the other hand, computational miRNA gene finding tools are strongly dependent on conservation criteria and other sequence/structure similarities with previously identified miRNA precursors which limits their power to identify novel miRNAs, particularly those which are not conserved [4].

Single-genome approaches are increasingly necessary given the fact that a growing number of genome sequencing projects are under way for which no evolutionarily close genome is available and for which one cannot otherwise hope to thoroughly explore the miRNA landscape.

Considering that we have but rudimentary models of miRNA precursor evolution, which makes it hard to interpret the biological significance of conservation data, and that we lack a deep understanding of the structural requirements for efficient pre-miRNA processing, we believe that if we are to increase our knowledge of the miRNA repertoire of an organism, our efforts should privilege general properties that are known to characterise miRNA precursors. These properties should not necessarily emerge from rules learnt from the detailed analysis of previously known precursors, but should rather focus on features that, in principle, distinguish pre-miRNAs for other hairpins.

In this paper, we propose a method to score pre-miRNA candidates from a single genome by combining four robustness/stability measures that are known to distinguish miRNA precursors from other genomic stem-loops. We use these measures to greatly reduce the initial set of candidates and we show that it consists of a high-sensitivity approach which is able to recover known miRNA precursors in *A. gambiae *and *D. melanogaster*. Furthermore, we apply our method to the newly sequenced genome of *Anopheles darlingi *where we show that our combined score (*cscore*) performs well by identifying 44 clear homologs of known pre-miRNAs in *Anopheles gambiae *amongst the top-scoring candidates. In addition, our approach is compared to a well-known single-genome method - Triplet-SVM [7] - and to a recent and sophisticated HHMM-based classification approach - HHMMiR [8].

This work is part of a framework under development, named CRAVELA http://www.cravela.org, which purports to identify and evaluate miRNA regulatory modules relying on heterogeneous sources of data.

## Results and Discussion

### The precursor candidate enumeration method recovers known pre-miRNAs

#### Most known pre-miRNAs are matched by precursor candidates

In order to evaluate the sensitivity of this procedure, it is necessary to assess whether known precursors are found amongst the extracted candidates. The number of known precursors and precursor candidates extracted from the datasets using the enumeration procedure described in the Methods section are shown in Table 1.

**Table 1:** Positive/Negative set sizes

Dataset	Positive Set	Negative Set	Overlapping	Extracted candidates
*A. gambiae*	67	2 244 922	92	2 245 014

*D. melanogaster*	157	1 316 105	200	1 316 305

*A. darlingi*	44	1 748 087	66	1 748 153

The Table shows the number of elements in the positive and negative sets, the number of candidates overlapping elements of the positive set and the total number of extracted candidates for each dataset. For *A. darlingi*, the positive set is the set of clear homologs to *A. gambiae *pre-miRNAs whereas in all other datasets the positive set corresponds to the known precursors. In all cases, the negative set consists in the presumptive non-precursor candidates which do not overlap precursors in the positive set.

Annotated sequences may or may not include sequences flanking the pre-miRNAs because the precise coordinates of the precursor hairpins are not always experimentally determined. The stem-loops identified by our enumeration strategy are extended stem-loops in the sense that they are the largest stem-loops contained in their local genomic contexts, and will therefore tend to be larger than precursor hairpins. In both cases, if the flanking sequences are short with respect to the actual precursor, the impact on the candidate evaluation procedure is likely to be modest.

Only very few known precursors are not matched by any candidate (1 out of 67 in *A. gambiae*, and 6 out of 157 in *D. melanogaster*). Since the enumeration procedure can only identify canonical stem-loops, some of these cases refer to multi-loop structures that share a common stem but with relatively small secondary stem-loops which fail to pass the minimum length criterion. Others are short structures which are filtered by the -20 kcal/mol stability criterion (see description of the enumeration procedure in the Methods section).

Additionally, the vast majority of annotated precursors with known mature sequences has a best match which includes the mature sequence in one of its stem arms (59 out of 67 in *A. gambiae*, and 134 out of 152 in *D. melanogaster*). It is worth to point out that in most cases a best match noted as not including the mature sequence in the stem arm in fact only misses the start or end of the mature sequence by a few nucleotides (beyond a 2-nt tolerance) because of a missed dangling end.

### A combination of measures scores *bona fide *pre-miRNAs above most precursor candidates

The measures used in this paper purport to assess whether the candidate precursors possess certain features that have been shown to distinguish pre-miRNAs from other stem-loops and are related to their stability and robustness.

It was shown that miRNAs have an *adjusted minimum free energy *(AMFE) that is lower than that of other stem-loop structures [9], i.e., when normalised for length, other genomic stem-loops tend to be less stable than miRNA precursors. Similarly, it was established that miRNA precursors tend to preserve roughly the same secondary structure in the face of variations in their genomic context [10], presumably as an evolved robustness to mutations in their flanking sequences (*Robustness to context*). Likewise, it was shown that miRNA precursor structures are usually also robust with respect to mutations (*Robustness to mutations*) [11], possibly as a result of second-order evolutionary processes. To these three measures, we add the requirement for *Robustness of Folding *observing that a true miRNA precursor should fold into a stable stem-loop structure for the most part of the structures in the thermodynamic ensemble where the molecule is found in physiological conditions.

From the combination of these four measures, it is possible to derive a single score for each precursor candidate and rank the stem-loops extracted from the datasets. Our single score not only combines the information provided by each measure, but it does so against a background of hairpins extracted from a random sequence with the same dinucleotide distribution of the original genome. This procedure compensates for hairpin robustness provided by genome composition alone.

In order to separate the problem of correctly identifying candidate precursors with that of assessing the performance of our evaluation measures, we take the known pre-miRNAs in each dataset as the positive set, and the negative set is made of all the candidates which do not overlap the coordinates of known precursors. The number of elements in the positive/negative sets for each dataset is shown in Table 1. The negative sets may include several yet unidentified precursors whose identification would have an impact on our performance assessment. To mitigate this problem and to assess the stability of the cutoff value for each measure as well as the combined score, and, more importantly, to deal with the greatly uneven positive/negative set sizes, we have adopted an undersampling procedure for the negative sets. In this procedure, we randomly extract 1 000 samples from the negative set each having the size of the positive set.

#### The combined score performs better than any individual measure

**Figure 1:**
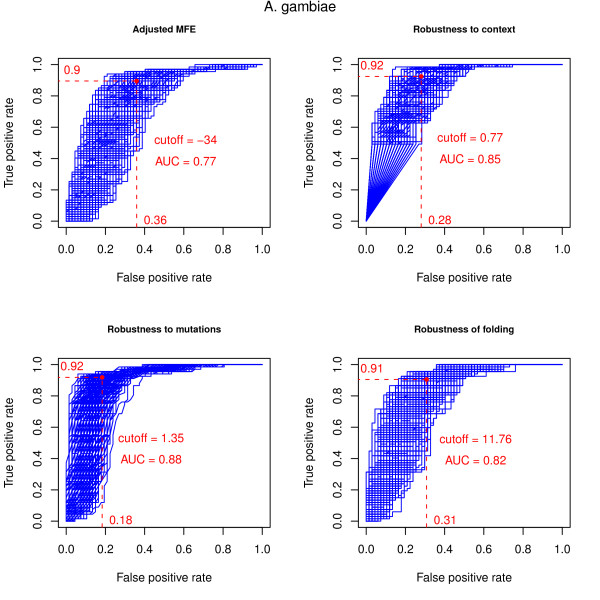
**ROC curves of the evaluation measures in the *A. gambiae *dataset**. ROC curves for the evaluation measures in the *A. gambiae *dataset. The dashed lines indicate the true/false positive rates for the average optimal cutoff, i.e., the average cutoff value that maximises the difference between true and false positive rates (TPR = TP/(TP + FN), FPR = FP/(FP + TN)). The negative sets consist of 1000 samples, each of the size of the positive set, drawn from the non-overlapping candidates. The average optimal cutoff value and the average AUC (area under the curve) value are also shown.

**Figure 2:**
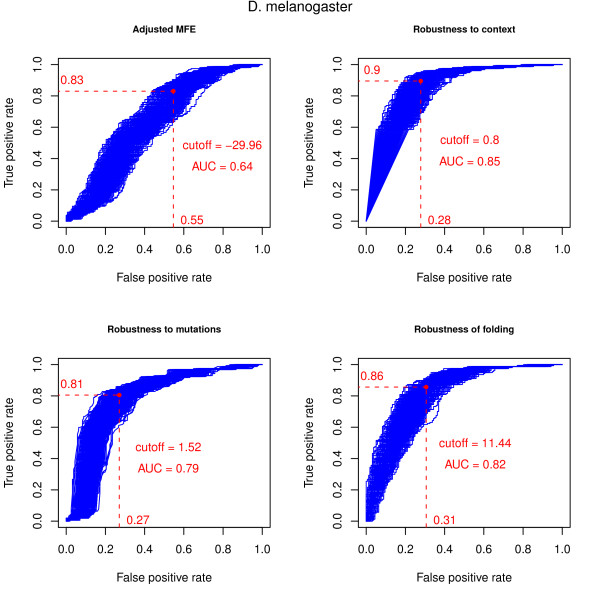
**ROC curves of the evaluation measures in the *D. melanogaster *dataset**. ROC curves for the evaluation measures in the *D. melanogaster *dataset. The dashed lines indicate the true/false positive rates of the average optimal cutoff, i.e., the average cutoff value that maximises the difference between true and false positive rates (TPR = TP/(TP + FN), FPR = FP/(FP + TN)). The negative sets consist of 1000 samples, each of the size of the positive set, drawn from the non-overlapping candidates. The average optimal cutoff value and the average AUC (area under the curve) value are also shown.

Figures 1 and 2 and Table 2 show the performance of the evaluation measures for the *A. gambiae *and *D. melanogaster *datasets, respectively.

**Table 2:** Sensitivity/Specificity

Dataset	Avg Optimal Cutoff	Sensitivity	Specificity
*A. gambiae*	0.41	90%	88%

*D. melanogaster*	0.3	83%	80%

*A. darlingi*	0.32	89%	84%

The average optimal cutoff value for each dataset over the 1 000 samples, alongside the average sensitivity and specificity values at that cutoff level.

The AMFE [9] measure performs best in the *A. gambiae *dataset. The fact that this measure does not compensate for GC content, which has a significant impact on folding free energy values, may explain the disparity of the results. The *D. melanogaster *dataset includes both euchromatic and heterochromatic sequences with different GC content, the latter having considerably lower values. The procedure used to combine the evaluation measures partially compensates for the lack of GC content normalisation because the randomised dataset is generated maintaining the same dinucleotide distribution of each of the original sequences. Replacing the AMFE with a modified version of the MFEI [9] measure, which does compensate for GC content and is discussed in the Methods section, had no discernible impact on the combined score (data not shown).

In both the *A. gambiae *and *D. melanogaster *datasets, the Robustness of Folding and the Robustness to Context measures have comparable performances in terms of average AUC, which summarises the relation between the true/false positive rates across all possible cutoff values for each of the samples of the negative set.

The Robustness to Mutations measure performs well with the *A. gambiae *dataset but the performance in the *D. melanogaster *dataset is negatively influenced by the presence of several long inverted repeats (mainly due to the inclusion of heterochromatic sequences) that are resilient to point mutations and thus attain a high score for this measure. These sequences should not be summarily excluded as they can include true miRNA precursors.

**Figure 3:**
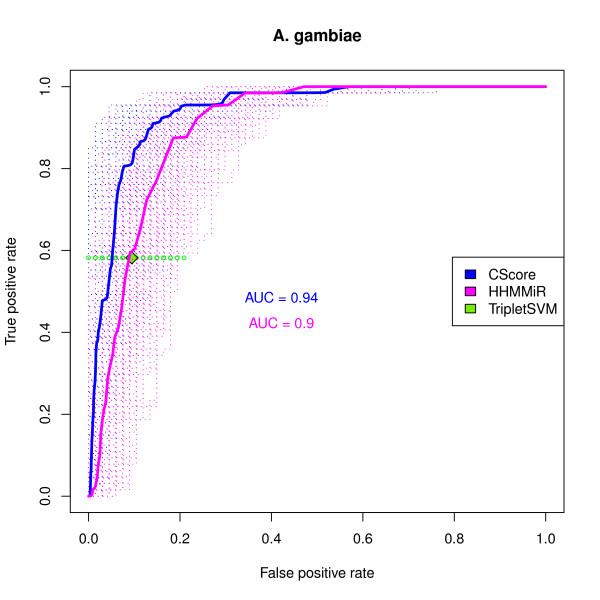
**ROC curve of the combined score in the *A. gambiae *dataset**. ROC curves for the *cscore *and HHMMiR in the *A. gambiae *dataset. The negative sets consist of 1000 samples, each of the size of the positive set, drawn from the non-overlapping candidates. The dashed lines are the individual ROC curves for each sample. The solid lines are the average ROC curves. The average AUC (area under the curve) values are also shown. The green diamond represents the average performance of the triplet-SVM pre-miRNA classifier and the smaller green circles represent its performance on each sample.

**Figure 4:**
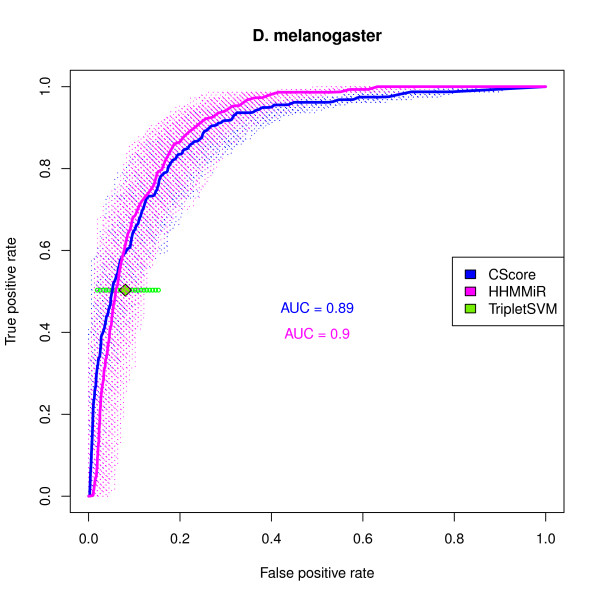
**ROC curve of the combined score in the *D. melanogaster *dataset**. ROC curves for the *cscore *and HHMMiR in the *D. melanogaster *dataset. The negative sets consist of 1000 samples, each of the size of the positive set, drawn from the non-overlapping candidates. The dashed lines are the individual ROC curves for each sample. The solid lines are the average ROC curves. The average AUC (area under the curve) values are also shown. The green diamond represents the average performance of the triplet-SVM pre-miRNA classifier and the smaller green circles represent its performance on each sample.

The results for the combined score (*cscore*) for both datasets are shown in Figures 3, and 4. In both cases, the *cscore *performs better than any individual measure in terms of average AUC, which means that, in general, for the same false positive rate (FPR = FP/(FP + TN)) one can attain higher sensitivity (Sensitivity = TPR) or, conversely, for the same true positive rate (TRP = TP/(TP + FN)) one can expect better specificity (Specificity = TN/(TN + FP) = 1 - FPR).

If we take the average optimal cutoff value for the *cscore *on each dataset, we obtain a reduced set of 328 829 candidates for *A. gambiae *and 287 469 for *D. melanogaster*. If we further eliminate all candidates whose genomic locations are overlapped by other candidates with higher *cscore*, the total number of candidates is reduced to 290 133 for *A. gambiae *and 240 751 for *D. melanogaster*.

#### The combined score performs well compared to other methods

Only a few classification methods can be readily compared to our combination of measures due to both the lack of use of conservation information and the need to evaluate a large number of candidates.

Triplet-SVM [7] is a fast and well-known binary classification method that uses a support vector machine (SVM) to learn sequence/structure features of pre-miRNAs in order to distinguish them from other genomic stem-loops. The feature vector used to train the SVM considers the pairing states of every three nucleotides (triplet) plus the identity of the nucleotide at the middle. The results presented here were obtained using the method with default parameters and the SVM model provided by the authors. Being a binary classification method, Triplet-SVM cannot be used to generate ROC curves for a direct comparison with our method. HHMMiR [8] is a sophisticated method based on hierarchical hidden-Markov models.

This method tries to learn the distinctive sequence/structure characteristics of different regions of the miRNA precursor. HHMMiR scores each candidate by calculating the ratio of the log-likelihoods generated by the positive and negative models (learnt from known pre-miRNAs and random hairpins, respectively).

Unlike with Triplet-SVM, the fact that HHMMiR can associate a score with each candidate elicits a direct comparison with our approach using ROC curves. Like before, the results presented for HHMMiR were obtained using default parameters and the maximum likelihood models provided by the authors.

The results presented in Figures 3 and 4 show the comparative performance of the *cscore*, Triplet-SVM and HHMMiR. The graphs illustrate the fact that Triplet-SVM tends to sacrifice sensitivity in order to obtain better specificity. In all datasets, the average performance of *cscore *always outperforms the average performance attained by Triplet-SVM.

The performances of the *cscore *and HHMMiR are quite similar in terms of average AUC. The *cscore *slightly outperforms HHMMiR for the *A. gambiae *dataset, whereas the reverse is seen in *D. melanogaster*. It is nonetheless surprising that a scoring scheme such as *cscore*, which makes no prior assumptions about precursor stem-loops except that they ought to be stable and robust, exhibits a performance comparable to a classifier that has been trained on known pre-miRNAs and is capable of sophisticated modelling of precursor sequences.

Both Triplet-SVM and HHMMiR are supervised learning methods which rely on training sets to produce a decision rule. In both cases, their ability to find novel miRNAs is dependent on how representative positive and negative examples turn out to be. The results presented here show that an approach that requires no prior training performs as well as the best of the two methods.

### Exploration of precursor candidates in *A. darlingi*

Our candidate enumeration and evaluation procedures were applied to the newly sequenced *A. darlingi*. A total of 1 748 153 precursor candidates were identified as shown in Table 1. To test our approach on a non-annotated genome, we analyse the performance of our *cscore *on three groups of candidates: those that are identified as homologs of known precursors from *A. gambiae*, those that are conserved in both genomes (excluding the homologs), and those which show low or no conservation (see the Methods sections).

We found clear homologs of 44 *A. gambiae *pre-miRNAs supported by both high-quality mutually best alignments and the observation that in all cases the mature sequence is perfectly conserved. The list of homologs and the alignment of the mature sequence with the homologous precursors is shown in the Supplementary Materials. The number of homologs identified corresponds to 67% of the pre-miRNAs known in *A. gambiae*, which is the closest sequenced genome to that of *A. darlingi*. All remaining known precursors in *A. gambiae *except one, despite not having clear homologous precursor sequences, do have identical mature sequences within the stem-arm of a precursor candidate in *A. darlingi*, which could indicate homology through conservation at a structural level. Additionally, we identified 7 855 precursor candidates conserved in both genomes (see Methods section).

The analysis of the distribution of the *cscore *for the three groups of candidates shows that the median scores are 0.613, 0.034, and 0.033 for the homologs, conserved and non-conserved candidates, respectively. The conserved and non-conserved stem-loops have similar *cscore *distributions, but the scores for the set of homologs, however, are distinctively higher. The fact that the score distribution for conserved and non-conserved candidates is very similar reinforces the idea that conservation criteria alone are not sufficient to identify good precursor candidates.

**Figure 5:**
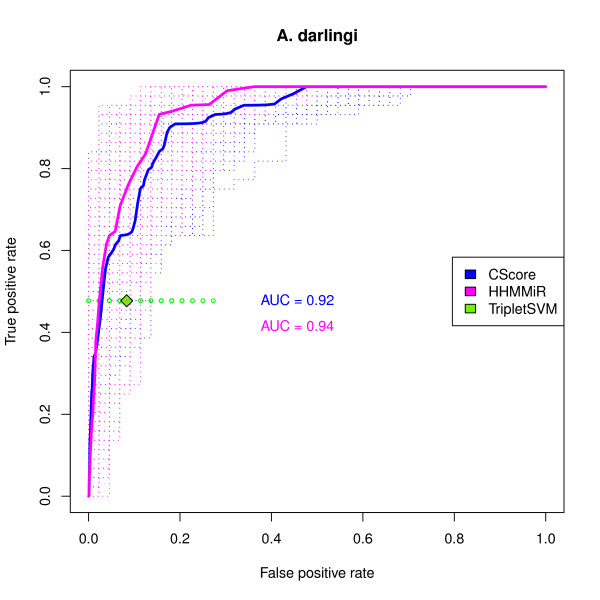
**ROC curve of the combined score in the *A. darlingi *dataset**. ROC curves for the *cscore *and HHMMiR in the *A. darlingi *dataset. The positive set consists of 44 clear homologs to *A. gambiae *pre-miRNAs. The negative sets consist of 1000 samples, each of the size of the positive set, drawn from the non-overlapping candidates. The dashed lines are the individual ROC curves for each sample. The solid lines are the average ROC curves. The average AUC (area under the curve) values are also shown. The green diamond represents the average performance of the triplet-SVM pre-miRNA classifier and the smaller green circles represent its performance on each sample.

Figure 5 shows the ROC curve for the performance of the *cscore *in the *A. darlingi *dataset using the pre-miRNA homologs as the positive set. The performances of Triplet-SVM and of HHMMiR are also shown. The results replicate what was observed in the other datasets. The *cscore *again outperforms Triplet-SVM and performs only slightly worse than HHMMiR.

There are 305 681 candidates above the average optimal cutoff for the *A. darlingi *dataset, which can be reduced to 248 970 by eliminating candidates overlapped by candidates with higher scores. Of these, 422 are found amongst the list of candidates conserved with respect to *A. gambiae*.

## Conclusions

Common computational strategies to address the problem of miRNA gene discovery usually involve the identification of a set of candidates that are subsequently filtered by their similarity with previously known pre-miRNAs and their degree of conservation in close species.

Although these approaches have vastly expanded the list of known miRNAs, relying merely on our general knowledge of the miRNA silencing pathway and arguments of similarity and conservation, they generally fail to integrate and use a growing amount of knowledge about these regulators. More significantly, these methods have introduced a strong bias favouring pre-miRNAs exhibiting extensive conservation and sharing a number of features with previously identified precursors. To varying degrees, their ability to discover non-conserved or non-canonical pre-miRNAs is therefore greatly reduced if not completely suppressed. This is not to say that one expects pre-miRNA features to vary greatly, but rather that the bias may not reveal the adequate learning set.

The method presented in this paper is part of a framework named CRAVELA which purports to be able to draw from heterogeneous sources of data and to offer an evidence-based tool to estimate the likelihood that a given set of precursor candidates are indeed pre-miRNAs. Here we have restricted our analysis to the portion of the framework responsible for extracting and sieving through a set of precursor candidates originating from a genome-wide search.

In this paper we show that by combining different robustness and stability measures, one can obtain a scoring scheme that outperforms any individual measure. There is, however, room for improvement. The procedure used to combine the different measures for each dataset relies on the measure distributions calculated for stem-loops extracted from artificial sequences generated with the same dinucleotide distributions observed in the original genome. As suggested by the relatively poorer results obtained for the *D. melanogaster *dataset, which includes both euchromatic and heterochromatic sequences, genome heterogeneity may warrant the generation of multiple artificial sequences with their respective measure distributions in order to obtain a scoring scheme able to cope with variations in sequence features for different regions of the genome.

The combination of measures that we present in this paper shows that there is a strong bias towards robust miRNA precursors and that this information can be used to reduce the vast number of stem-loops that are found in metazoan genomes. Nonetheless, given the number of precursor candidates that remain after sieving through those which were identified in the three datasets we have presented, one cannot claim that we are in the presence of a miRNA gene finding method. We have, however, reduced the number of candidates by one order of magnitude, without resorting to classic approaches and retaining most known pre-miRNAs in each dataset. The main advantage of the adopted score is that it relies on intelligible criteria based on arguments of biological plausibility. For annotated genomes, further reductions of the number of candidates can readily be used, namely by eliminating all candidates overlapping regions with annotations incompatible with the presence of a pre-miRNA. For genomes that have transcription data made available, that information can be used to restrict the candidates to those for which there is evidence of transcription.

Over this reduced set of candidates one can now apply any number of structure/sequence features analyses, conservation criteria, or other filters depending only on the information that is available for the genome under study.

## Methods

### Data preparation

The genome sequences for the chromosomal arms X, 2R, 2L, 3R, 3L and UNKN of *A. gambiae *(assembly AgamP3) were obtained from the Ensembl ftp site ftp://ftp.ensemblgenomes.org/pub/metazoa. Both the euchromatic and heterochromatic genomic sequences of *Drosophila melanogaster *(release 5) were obtained from the BDGP project website http://www.fruitfly.org/sequence/release5genomic.shtml. All the sequences concerning known pre-miRNAs and their respective mature sequences were recovered from the miRBase webserver (http://www.mirbase.org, release 13).

The Whole Genome Shotgun project of the newly sequenced *A. darlingi *has been deposited at DDBJ/EMBL/GenBank under the accession ADMH00000000. The version described in this paper is the first version, ADMH01000000. It consists of 18 629 contigs with a total size of 173 473 443 nt. The results presented in this paper have been made available on our framework website http://www.cravela.org.

### Implementation

The algorithm to identify potential stem-loops in a given genome is implemented in C. Two additional PERL programs perform the folding of the candidate regions and filter candidates by size and folding energy while only keeping the longest candidates for the same terminal loop coordinates. The folding script is able to run on multiple program instances to take advantage of multi-core machines.

PERL programs are used to load genomic information to a MySQL database, onto which all information about candidate precursors is also loaded. Additional PERL programs compute each evaluation measure described in this paper and these programs are implemented in such a way as to allow the entire candidate set to be partitioned into independent batches rendering them amenable to parallel processing by a computer cluster.

All graphs in this paper are made using R scripts which in turn are automatically generated by PERL programs.

The code developed for this project requires PERL 5.8.6 or higher, MySQL 5.5 or higher, R 2.10.1 or higher, and ViennaRNA 1.6.5 or higher.

The latest version of the programs is available at http://www.cravela.org/ and the current version is also made available as additional files 1, 2, 3, 4, 5 and 6.

The authors add no restrictions to use by non-academics other than those in place for the required software referred to above.

### Efficient identification of candidate hairpins

Candidate hairpins are identified by sliding a 200-nt window across the genome and using a dynamic programming function to evaluate the pairing potential of the two halves of the window (see Supplementary Materials for further details). Only local maxima of this evaluation function are considered candidate windows and contiguous windows yielding the same score are merged into the same candidate region. This filtering procedure reduces the number of candidate regions to consider to less than 1/10 of the total initial number of windows (data not shown).

Having identified the candidate regions, these are folded using RNAfold with standard parameters and the largest hairpin structure found is extracted and re-folded. The final set of precursor candidates is made up of these refolded stem-loops restricted to those which exhibit a minimum free energy no higher than -20 Kcal/mol and with both stem arms at least 16-nt long, since these parameters will capture the vast majority of known pre-miRNAs while significantly reducing the number of candidate stem-loops. The set of candidates is subjected to an additional filtering step in order to identify different candidates with identical terminal loop coordinates in which case only the longest candidate is retained.

### Evaluation measures

#### Adjusted MFE

The Adjusted MFE (AMFE) is defined as:

$$\text{AMFE}=\text{100}\frac{\text{MFE}}{\text{length}}$$

This measure consists in normalising the minimum free energy of the structure under study with respect to its length. The normalisation procedure is justified by the observation that larger structures can have lower free energies due simply to the fact that they have more opportunities to form base-pairs. GC content also plays a role in defining the lower limit of free energy a structure can exhibit, but additional normalisation by GC content as done in [9] using the MFEI (Minimum Free Energy Index) yields an ill-defined measure for candidates from AT-rich regions which will sometimes have a GC content of zero. The procedure used to calculate the *cscore*, described below, partially compensates for the absence of this normalisation step.

#### Robustness of folding

This measure refers to the fraction of base-pairs that are preserved across a set of sub-optimal structures and is implemented by RNAfold as a measure of ensemble diversity [12].

The value of this measure is the average base-pair distance between the optimal and each of the sub-optimal structures in the ensemble weighted according to the following formula:

$$\langle d_{\text{bp}}\rangle =\sum_{i}d_{\text{bp}}\left(s_{0},s_{i}\right)\frac{e^{-\Delta G_{i}/kT}}{Z}$$

where *s*_0 _is the optimal structure and *s*_i _is the *i*th sub-optimal structure. The computed value is then normalised for a sequence with a length of 100-nt.

#### Robustness with respect to context

Given the large number of candidates and the need for computational efficiency, instead of strictly preserving dinucleotide frequencies, as proposed in [10], we trained two single-state first-order hidden-Markov models with the up- and downstream genomic contexts of each candidate, covering a length identical to the size of the candidate. The candidates are then re-folded in 100 random contexts generated according to the hidden-Markov models previously obtained. The value of this measure is the median proportion of the original structure that is preserved in each refolded candidate. The measure takes values between 0 (none of the original structure is preserved) and 1 (the entire structure remains intact).

#### Robustness with respect to mutations

For each candidate precursor, the entire 1-mutation neighbourhood is generated and each mutant is folded. The value of this measure is the median base-pair distance between the original and each mutant structure, normalised to a sequence with a length of 100-nt.

The authors who proposed the measures of robustness with respect to context and robustness with respect to mutations, discussed above, calculated their scores by averaging the proportion of preserved structure and base pair distance to each mutant, respectively. We, instead, preferred the median of these values since it is a more robust centrality measure when dealing with possibly non-Gaussian distributions.

### Combining measures

In order to combine the measures described above into a single score, we determine the significance of the value of the measures for each candidate against its empirical distribution in a random genome with similar dinucleotide frequencies. To that effect, a single-state first-order hidden-Markov model is trained with the sequences of the genome whose candidates are to be evaluated, which is then used to generate a single 5 Mb sequence. Precursor candidates are extracted from these artificial genomes and evaluated using each of the four measures.

The *cscore *of a precursor candidate is given by the product of the significance of each measure value against the respective empirical distribution in the artificial genome, i.e., the proportion of candidates in the artificial genome which have worse scores than the candidate under consideration. This notion of worse can either mean lower or higher values, depending on the biological interpretation of the measure.

The *cscore *thus varies between 0 (the candidate scores worse than all candidates in the artificial genome for at least one measure) and 1 (the candidate scores better than all candidates in the artificial genome for all measures).

### Identification of homologs and conserved stem-loops

Pre-miRNA homologs were found by performing a **Blastn **search and identifying a two-way best alignment with respect to the set of known pre-miRNAs of *A. gambiae *and the set of candidates from *A. darlingi*. Only homologous sequences that folded into stem-loops with MFE < -20 Kcal/mol and with both stem arms longer than 16-nt were considered homologous precursors. Conserved stem-loops were also determined by a two-way best alignment between the candidates of both genomes, restricted to alignments with E-value below 1e - 20 and excluding all *A. gambiae *candidates that overlap known precursors.

## Authors' contributions

NDM developed the software, performed the analyses, contributed to the design of the approach and wrote the manuscript. ATF, MFS, and ATV contributed to the design of the approach and helped draft the manuscript. ATV made available the data for the *A. darlingi *dataset. All authors read and approved the final version of the manuscript.

## Supplementary Material

Additional file 1 **Extractor**. Source code for C program which identifies candidate regions potentially containing stem-loops in a dataset of interest. Source code for PERL programs which identify and filter stem-loops within the extracted candidate regions. Click here for file

Additional file 2 **Loading information onto database**. Source code for PERL programs that load precursor information onto the database. Click here for file

Additional file 3 **Computing evaluation measures**. Source code for PERL programs that compute the evaluation measures and calculate the composite score. Click here for file

Additional file 4 **Common PERL modules**. PERL Modules shared across all PERL programs. Click here for file

Additional file 5 **Generating graphs**. PERL programs which generate R scripts that calculate and draw graphs. Click here for file

Additional file 6 **Supplementary Materials**. Detailed presentation of the candidate enumeration procedure. Table showing the homologs to pre-miRNAs of *A. gambiae *identified amongst the precursor candidates of *A. darlingi*. Table showing the alignments of mature miRNAs from *A. gambiae *with precursor homologs identified amongst precursor candidates from *A. darlingi*. Click here for file
