# Supplementary material for: Combination of measures distinguishes pre-miRNAs from other stem-loops in the genome of the newly sequenced Anopheles darlingi
Source: BMC Genomics. 2010 Sep 29;11:529. doi: 10.1186/1471-2164-11-529 (PMC2997018; doi:10.1186/1471-2164-11-529)
Supplement: Additional file 6 — Supplementary Materials. Detailed presentation of the candidate enumeration procedure. Table showing the homologs to pre-miRNAs of A. gambiae identified amongst the precursor candidates of A. darlingi. Table showing the alignments of mature miRNAs from A. gambiae with precursor homologs identified amongst precursor candidates from A. darlingi. [file 1471-2164-11-529-S6.PDF]

# Combination of measures distinguishes pre-miRNAs from other stem-loops in the genome of the newly sequenced *Anopheles darlingi*

Nuno D. Mendes*[1,2,3], Ana T. Freitas[2], Ana T. Vasconcelos[4], Marie-France Sagot[1,3]

[1]Équipe BAOBAB, Laboratoire de Biométrie et Biologie Évolutive (UMR 5558); CNRS; Univ. Lyon 1, 43 bd du 11 nov 1918, 69622, Villeurbanne Cedex, France
[2]IST/INESC-ID, 9 Rua Alves Redol, 1000-029 Lisbon, Portugal
[3]BAMBOO Team, INRIA Rhône-Alpes, 655 avenue de l'Europe, 38330 Montbonnot Saint-Martin, France
[4]Bioinformatics Laboratory, National Laboratory of Scientific Computation (LNCC), Avenida Getúlio Vargas, 333, Petrópolis, Brazil

Email: Nuno D. Mendes*- ndm@kdbio.inesc-id.pt; Ana T. Freitas - atf@kdbio.inesc-id.pt; Ana T. Vasconcelos - atrv@lncc.br; Marie-France Sagot - marie-france.sagot@inria.fr;

*Corresponding author

## Supplementary Materials

### Efficient identification of candidate hairpins

The problem of identifying candidate stem-loop structures in a genome can be cast as the problem of finding an imperfect palindrome with a central intervening sequence. Scanning the entire genome of interest in an attempt to seek imperfect palindromes directly proved to be computationally unfeasible, especially considering the irregular nature of these stem-loop structures with potentially numerous and large bulges as well as non-canonical base pairings.

We adopted, instead, a filtering approach based on the observation that a segment of a genome which upon transcription can adopt a stem-loop conformation should exhibit a higher degree of potential pairing between its two halves (if the midpoint of the segment falls within the region corresponding to the terminal loop) than a segment that either does not contain a stem-loop or only partially contains such a structure.

Let $S$ be a string over an alphabet $\Sigma$, $|S|$ denotes the length of the string, and $S_i$ denotes the $i$th position of the string, with $0 < i \leq |S|$, $\overleftarrow{S}$ denotes the reversed string, i.e., $\overleftarrow{S} = S_{|S|} \ldots S_1$. We denote by $L^S = S_1 \ldots S_{\lfloor |S|/2 \rfloor}$ and $R^S = S_{\lceil |S|/2 \rceil} \ldots S_{|S|}$, respectively, the left and right halves of string $S$ so that $S = L^S R^S$.

Let $\Phi \subset \Sigma^2$ be the set of accepted pairings of characters in $\Sigma$. The set $\Phi$ induces the predicate $F_\Phi : \Sigma^* \times \Sigma^* \mapsto \{0,1\}$ defined as follows: $F_\Phi(a\alpha, b\beta) = 1 \quad \text{iff} \quad (a,b) \in \Phi \wedge (F_\Phi(\alpha,\beta) = 1 \quad \vee \quad \alpha = \beta = \varepsilon)$, with $a, b \in \Sigma$, $\alpha, \beta \in \Sigma^*$, and $\varepsilon$ being the empty string.

In order to consider acceptable RNA pairings, including all canonical pairs along with G:U basepairs, we have $\Phi = \{(\texttt{A},\texttt{U}), (\texttt{U},\texttt{A}), (\texttt{C},\texttt{G}), (\texttt{G},\texttt{C}), (\texttt{G},\texttt{U}), (\texttt{U},\texttt{G})\}$.

The best local alignment over a split string $S$ is calculated using a DP matrix where each cell, $H(i,j)$, is computed with the following recurrence:

$$\max \left\{ \begin{array}{ll} 0 & \\ H(i-1,j-1) + \xi_0 & \text{if } F_\Phi({L^S}_i, {\overleftarrow{R^S}}_j) = 1 \\ H(i-1,j-1) - \xi_1 & \text{if } F_\Phi({L^S}_i, {\overleftarrow{R^S}}_j) = 0 \\ H(i-1,j) - \xi_2 & \\ H(i,j-1) - \xi_2 & \end{array} \right\}$$

where $\xi_0$, $\xi_1$, and $\xi_2$ represent the contribution of matches, mismatches, and gaps, respectively, to the alignment score.

Using the Smith-Waterman algorithm on a split string, $S$, one can determine the best alignment in $O(|S|^2)$.

Consider a genome with $k$ chromosomes, seen as a collection of sequences $\mathcal{S} = \{S_1, S_2, \ldots, S_k\}$. The algorithm will slide a window of length $w$ along each chromosome of the genome determining, for each position, the best local alignment under a model $M = (\xi_0, \xi_1, \xi_2)$.

Using the described sliding-window procedure, the best alignments for all windows in the genome can be computed in $O(w^2 \sum_i (|S_i| - w + 1))$.

An alignment $\Lambda$ of two sequences $S_1, S_2$ is a tuple $(e_1, e_2, \sigma)$ where $0 \le e_1 \le |S_1|$, $0 \le e_2 \le |S_2|$, and $\sigma \in \{\uparrow, \leftarrow, \nwarrow\}^*$.

If $\Lambda = (e_1, e_2, \sigma)$ is a best local alignment of a split string, then:

- $\forall i, j \quad H(i,j) \le H(e_1, e_2)$, where $H(i,j)$ is the value of the $i$th row, $j$th column of the DP matrix of the Smith-Waterman algorithm.
- $\sigma$ represents a path from $(e_1, e_2)$ to a cell in the DP matrix containing the value 0 such that if the $k$th cell in the path is $(i_k, j_k)$ and $H(i_k, j_k) \neq 0$ then the $(k+1)$th cell in the path is:
  - $(i_k - 1, j_k - 1)$ if $H(i_k, j_k) = H(i_k - 1, j_k - 1) + \xi_0$ and $\sigma_k = \nwarrow$
  - $(i_k - 1, j_k - 1)$ if $H(i_k, j_k) = H(i_k - 1, j_k - 1) - \xi_1$ and $\sigma_k = \nwarrow$
  - $(i_k - 1, j_k)$ if $H(i_k, j_k) = H(i_k - 1, j_k) - \xi_2$ and $\sigma_k = \leftarrow$

– $(i_k, j_k - 1)$ if $H(i_k, j_k) = H(i_k, j_k - 1) - \xi_2$ and $\sigma_k = \uparrow$

The rationale of the procedure is to take the best alignment of the two halves of each genome window considered and identify the windows where the pairing potential is locally maximal with respect to a normalised score.

The normalised score for a best local alignment $\Lambda = (e_1, e_2, \sigma)$ of a split string is defined as:

$$s(\Lambda) = \begin{cases} \frac{2H(e_1,e_2)}{e_1+e_2} & \text{if } e_1 + e_2 > 0 \\ 0 & \text{otherwise} \end{cases}$$

The adopted score not only normalises the score of the best alignment with respect to the alignment length, but it also privileges a base pairing closer to the midpoint of the genome window under consideration. We can now define what is a candidate position in the genome.

Consider a chromosome of a given genome. Let $S_p$ be the sequence of length $w$ starting at position $p$ of the said chromosome, and let $\Lambda_p$ be the best local alignment in $S_p$. We have that $S_p$ is a candidate sequence iff the normalised score is locally maximal at $S_p$, i.e.,

1. $\exists \hat{p} : \forall p' : \hat{p} < p' \leq p \implies s(\Lambda_{\hat{p}}) < s(\Lambda_p) = s(\Lambda_{p'})$
2. $\exists \hat{p} : \forall p' : \hat{p} > p' \geq p \implies s(\Lambda_{\hat{p}}) < s(\Lambda_p) = s(\Lambda_{p'})$

having $s(\Lambda_{\hat{p}}) = 0$ for every $\hat{p} < 0$ or $\hat{p} > |S| - w + 1$.

As several candidate positions may be identified in contiguous co-ordinates in the genome, presumably for each window whose midpoint falls within the terminal loop portion of the stem-loop, we aggregate them together in candidate regions as they will refer to the same stem-loop structure.

Let $R_p^l$ be a region of length $l \geq w$ starting at position $p$ of a chromosome $S$ of a given genome. $R_p^l$ is a candidate region iff $S_p, \ldots, S_{p+l-w}$ are candidate positions and $S_{p-1}, S_{p+l-w+1}$ are not.

We have chosen a window length of 200, which approximately corresponds to the length of the largest annotated metazoan precursor sequence and is wide enough to accommodate the vast majority of known animal pre-miRNAs, and we have adopted a scoring model such that $\xi_0 = \xi_1 = \xi_2 = 1$. The choice of parameters for the model is important since it may affect the identity and amount of candidate regions identified. Our scoring model was based on three observations. First, most DNA alignment methods prefer a linear model for gaps and an equal penalty for gaps and mismatches [1]. Second, miRNA precursors necessarily exhibit gaps and mismatches when aligning their stem portion due to the ubiquitous yet small bulges and inner loops which justifies that the penalty for a gap/mismatch is the same as the contribution

of matches. Finally, small variations in the scoring model did not produce significantly different results, whereas more radical departs from the adopted model, such as having mismatches or gaps negatively contributing to the alignment score more than twice the contribution of a match, did have an impact on the sensibility (data not shown).

Having identified the candidate regions, these are folded using RNAfold with standard parameters and the largest stem-loop structure contained therein is extracted and re-folded. The final set of precursor candidates is made up of these refolded stem-loops restricted to those which exhibit a minimum free energy no higher than -20 Kcal/mol and with both stem arms at least 16-nt long, since these parameters will capture the vast majority of known pre-miRNAs while significantly reducing the number of candidate stem-loops. The set of candidates is subjected to an additional filtering step in order to identify different candidates with identical terminal loop co-ordinates in which case only the longest candidate is retained.

**Table 1 - Homologs to pre-miRNAs of *A. gambiae* identified amongst the precursor candidates of *A. darlingi***

The Table shows the homologs to pre-miRNAs of *A. gambiae* identified amongst the precursor candidate sequences of *A. darlingi*, their position in the *A. darlingi* dataset and the E-value and identity percentage of the `Blastn` hit.

Table 1:

| miRNA | Contig | Strand | Start | Stop | Length | E-value | Identity |
|---|---|---|---|---|---|---|---|
| **aga-mir-281** | ctg7180000455710 | F | 12304 | 12396 | 93 | 6e-44 | 98.92 |
| **aga-mir-137** | ctg7180000423045 | F | 24973 | 25062 | 90 | 3e-42 | 98.89 |
| **aga-mir-125** | ctg7180000436522 | R | 30069 | 30161 | 93 | 2e-41 | 97.85 |
| **aga-mir-9c** | ctg7180000436657 | F | 27474 | 27563 | 90 | 8e-40 | 97.78 |
| **aga-mir-iab-4** | ctg7180000409079 | R | 9295 | 9378 | 84 | 1e-38 | 98.81 |
| **aga-mir-278** | ctg7180000393996 | R | 2139 | 2222 | 84 | 3e-36 | 98.81 |
| **aga-mir-8** | ctg7180000296739 | R | 14269 | 14350 | 82 | 5e-35 | 97.56 |
| **aga-mir-957** | ctg7180000502071 | F | 10129 | 10209 | 81 | 2e-34 | 97.53 |
| **aga-mir-1175** | ctg7180000395096 | R | 20239 | 20316 | 78 | 1e-32 | 97.44 |
| **aga-mir-305** | ctg7180000409513 | R | 69 | 156 | 88 | 5e-32 | 95.51 |
| **aga-mir-9a** | ctg7180000394369 | R | 31986 | 32065 | 80 | 2e-31 | 97.50 |
| **aga-mir-79** | ctg7180000436657 | F | 29361 | 29431 | 71 | 8e-31 | 98.59 |
| **aga-mir-263b** | ctg7180000380439 | F | 18574 | 18666 | 93 | 8e-31 | 94.74 |
| **aga-mir-927** | ctg7180000364658 | R | 2214 | 2301 | 88 | 1e-29 | 94.32 |
| **aga-mir-1891** | ctg7180000436657 | F | 69525 | 69616 | 92 | 5e-29 | 92.39 |
| **aga-mir-1000** | ctg7180000409240 | F | 26262 | 26333 | 72 | 5e-29 | 97.22 |
| **aga-mir-929** | ctg7180000436895 | R | 35399 | 35472 | 74 | 7e-28 | 95.95 |
| **aga-mir-993** | ctg7180000380779 | R | 2989 | 3093 | 105 | 1e-27 | 90.57 |

Continued on Next Page...

| miRNA | Contig | Strand | Start | Stop | Length | E-value | Identity |
|---|---|---|---|---|---|---|---|
| **aga-mir-307** | ctg7180000501812 | F | 90845 | 90913 | 69 | 3e-27 | 97.10 |
| **aga-mir-7** | ctg7180000456148 | R | 10858 | 10934 | 77 | 3e-27 | 94.81 |
| **aga-mir-283** | ctg7180000394200 | R | 11363 | 11450 | 88 | 1e-26 | 92.05 |
| **aga-mir-14** | ctg7180000394624 | F | 29822 | 29905 | 84 | 1e-26 | 94.05 |
| **aga-mir-210** | ctg7180000325517 | R | 378 | 447 | 70 | 1e-25 | 95.71 |
| **aga-mir-92b** | ctg7180000502202 | R | 52014 | 52091 | 78 | 2e-25 | 93.59 |
| **aga-mir-190** | ctg7180000409440 | F | 26813 | 26893 | 81 | 8e-25 | 93.83 |
| **aga-mir-184** | ctg7180000395192 | F | 12643 | 12726 | 84 | 2e-24 | 94.05 |
| **aga-bantam** | ctg7180000380411 | F | 3920 | 4019 | 100 | 3e-24 | 93.07 |
| **aga-mir-263** | ctg7180000422962 | R | 6187 | 6272 | 86 | 2e-22 | 95.35 |
| **aga-mir-277** | ctg7180000436725 | F | 2857 | 2947 | 91 | 3e-21 | 90.11 |
| **aga-mir-124** | ctg7180000394913 | F | 3659 | 3737 | 79 | 9e-21 | 90.36 |
| **aga-mir-10** | ctg7180000299625 | F | 88710 | 88792 | 83 | 7e-19 | 89.16 |
| **aga-mir-13b** | ctg7180000296969 | F | 18851 | 18926 | 76 | 3e-18 | 92.21 |
| **aga-mir-988** | ctg7180000423020 | R | 24356 | 24424 | 69 | 3e-18 | 92.86 |
| **aga-mir-276** | ctg7180000394910 | R | 4307 | 4390 | 84 | 1e-17 | 89.41 |
| **aga-mir-219** | ctg7180000456051 | R | 64129 | 64209 | 81 | 4e-17 | 89.41 |
| **aga-mir-282** | ctg7180000297228 | F | 31847 | 31926 | 80 | 6e-16 | 90.12 |
| **aga-mir-9b** | ctg7180000436657 | F | 29840 | 29918 | 79 | 4e-14 | 87.21 |
| **aga-mir-1890** | ctg7180000297175 | R | 19896 | 19966 | 71 | 4e-14 | 90.14 |
| **aga-mir-317** | ctg7180000381136 | F | 4237 | 4319 | 83 | 2e-12 | 86.90 |
| **aga-mir-275** | ctg7180000409513 | R | 6089 | 6155 | 67 | 2e-12 | 89.71 |
| **aga-mir-87** | ctg7180000358126 | R | 296 | 383 | 88 | 1e-11 | 85.56 |
| **aga-mir-308** | ctg7180000296848 | R | 3648 | 3718 | 71 | 1e-11 | 88.73 |
| **aga-mir-279** | ctg7180000436869 | R | 24027 | 24088 | 62 | 2e-09 | 88.89 |
| **aga-mir-92a** | ctg7180000502202 | R | 73930 | 73990 | 61 | 9e-09 | 88.52 |

**Table 2 - Alignment of mature miRNAs from *A. gambiae* against precursor homologs identified amongst pre-miRNA candidates from *A. darlingi***

The Table shows the alignment of mature miRNAs from *A. gambiae* to the precursor homologs identified amongst the precursor candidate sequences of *A. darlingi*.

Table 2: Alignment of mature miRNAs from *A. gambiae* against precursor homologues identified amongst pre-miRNA candidates from *A. darlingi*

| miRNA | Alignment | Identity |
|---|---|---|
| **aga-mir-281** | 5′ AUCGAAUGUGAAAAUAAAGAGAGCUAUCCGUCGACAGUAGGGAUAUAAUUCACUGUCAUGGAAUUGCUCUCUUUAUGUACAAUUCGAUAUUCA 3′<br>5′ UGUCAUGGAAUUGCUCUCUUUAU 3′ | 100.00 |
| **aga-mir-137** | 5′ AAAACUUGGUUGGCCACGCGUAUUCUUGGGUUACUAACACACUAUUUAUGUUGUUAUUGCUUGAGAAUACACGUAGUUGACUAGUGUUGU 3′<br>5′ UAUUGCUUGAGAAUACACGUAG 3′ | 100.00 |
| **aga-mir-125** | 5′ GUAUCUGCUGAUUCCCUGAGACCCUAACUUGUGACUAUCGUUACAAAGUUUCACAAGUUUUGAUCUCCGGUAUUAGCGGUUGAGAUGCGACGG 3′<br>5′ UCCCUGAGACCCUAACUUGUGA 3′ | 100.00 |
| **aga-mir-9c** | 5′ UUUCCGGCUGUGUCUUUGGUAUUCUAGCUGUAGAAUGUUGUUUUGAUUGUAAUAUCUCUAAAGCUUUAGUACCAGAGGUCCAACUGGGAA 3′<br>5′ UCUUUGGUAUUCUAGCUGUAGA 3′ | 100.00 |
| **aga-mir-iab-4** | 5′ GUGCCGCUUCAUGAACGUAUACUGAAUGUAUCCUGAGUGCUACUUAUCCGGUAUACCUUCAGUAUACGUAACAGGAGGCGACAC 3′<br>5′ ACGUAUACUGAAUGUAUCCUGA 3′ | 100.00 |
| **aga-mir-278** | 5′ GGUACGGUACGGACGGACGAUAGUCUUCAACGACCGUUCACGUUUGACACGAGGUCGGUGGGACUUUCGUCCGUUUGUAAGGCC 3′<br>5′ UCGGUGGGACUUUCGUCCGUUU 3′ | 100.00 |
| **aga-mir-8** | 5′ GUCUGUUCACAUCUUACCGGGCAGCAUUAGAUAUUUUAUCGGAUACUUCUAAUACUGUCAGGUAAAGAUGUCGUCCGAGCCC 3′<br>5′ UAAUACUGUCAGGUAAAGAUGUC 3′ | 100.00 |
| **aga-mir-957** | 5′ ACUGCGGGCGUUAGUUUUGGGCGGGUUUUAGUGUAUUUCGAUGAGAAUUCUAUUGAAACCGUCCAAAACUGAGGCCGGCAG 3′<br>5′ UGAAACCGUCCAAAACUGAGGC 3′ | 100.00 |
| **aga-mir-1175** | 5′ GAUAUGGAAUAAGUGGAGUAGUGGUCUCAUCGCUUAGUUUUAGAAAAGUGAGAUUCUACUUCUCCGACUUAAUUCAUA 3′<br>5′ UGAGAUUCUACUUCUCCGACUUAA 3′ | 100.00 |
| **aga-mir-305** | 5′ UUUGUCACAUGUCUAUUGUACUUCAUCAGGUGCUCUGGUGGAUUUCAGAAACCCGGCACAUGUUGGAGUACACUUAAUGUGCUGACAA 3′<br>5′ AUUGUACUUCAUCAGGUGCUCUG 3′ | 100.00 |
| **aga-mir-9a** | 5′ GUCAAUGUUCUCUUUGGUUAUCUAGCUGUAUGAGUGUAUUUAAAAACGUCAUAAAGCUAGCAUACCGAAGUUAAUAAUUG 3′<br>5′ UCUUUGGUUAUCUAGCUGUAUGA 3′ | 100.00 |
| **aga-mir-79** | 5′ GCUUUGGCGCUUUAGCUGUAUGAUAGAAUUUGAAGUAUUUCAUAAAGCUAGAUUACCAAAGCAUAGACGAA 3′<br>5′ UAAAGCUAGAUUACCAAAGCAU 3′ | 100.00 |
| **aga-mir-263b** | 5′ UGACCAAUUAUGGACCUUGGCACUGGGAGAAUUCACAGUGAUCGUACAUUAUCGUUCUGUGGAUCUUUUCGUGCCAUCGUUCAAAUUUGGUGC 3′<br>5′ CUUGGCACUGGGAGAAUUCAC 3′ | 100.00 |
| **aga-mir-927** | 5′ GUUAAUGGUUCGUUUUAGAAUUCCUACGCUUUACCCGUUAAAUUAAAGUAGUGCGGCAAAGCGUUUGGAUUCUGAAACGAAACAUUAA 3′<br>5′ UUUAGAAUUCCUACGCUUUACC 3′ | 100.00 |
| **aga-mir-1891** | 5′ UCUUUUUCUGUCAUGUUGAGGAGUUAAUUUGCGUGUUUUUGCAUACGAUUAAACACGUCCAUUAACUCUGGUACAUGAUGGAAAAACCGAGC 3′<br>5′ UGAGGAGUUAAUUUGCGUGUUUU 3′ | 100.00 |

Continued on Next Page...

| miRNA | Alignment | Identity |
|---|---|---|
| **aga-mir-1000** | 5′ GUCGAUGAUAUUGUCCUGUCACAGCAGUACUAUUUGCCUAGCUUACUGUUGUUUCGGGACAUUUCCAUCGAC 3′<br>5′ AUAUUGUCCUGUCACAGCAGU 3′ | 100.00 |
| **aga-mir-929** | 5′ UGGGAUUAAAUUGACUCUAGUAGGGAGUCCUUCCAUACGAGAGACUCCCUAACGGAGUCAGAUUGAUUCCGGUA 3′<br>5′ CUCCCUAACGGAGUCAGAUUG 3′ | 100.00 |
| **aga-mir-993** | 5′ ...CGUGACCUACCCUGUAGUUCCGGGCUUUUGUGGGUUGAAAUACAAAAACAUGUAAAUUCAUAUUCUCUUAUCAGAAGCUCGUUUCUAUAGAGGUAUCUCA 3′<br>5′ GAAGCUCGUUUCUAUAGAGGUAUCU 3′ | 100.00 |
| **aga-mir-307** | 5′ UCUCUCGAUUACUCACUCAACCUGGGUGUGAUGCUUAUUUGAAUCAUCACAACCUCCUUGAGUGAGCGA 3′<br>5′ UCACAACCUCCUUGAGUGAG 3′ | 100.00 |
| **aga-mir-7** | 5′ UUGUAUGGAAGACUAGUGAUUUUGUUGUUUGGCUUAAGAUACUAACAAUAAAUCCCUUGUCUUUCUACAAAGAUUGC 3′<br>5′ UGGAAGACUAGUGAUUUUGUUGU 3′ | 100.00 |
| **aga-mir-283** | 5′ UUCGACUGAAAGGUAAAUAUCAGCUGGUAAUUCUAGGCUAUCUAAACUUCGUGCACCCCGGAAUUUCAGCUGAUAUCCACUUUUCCGU 3′<br>5′ UAAAUAUCAGCUGGUAAUUCU 3′ | 100.00 |
| **aga-mir-14** | 5′ GCCCGAUAAGCCUGUGGGAGCGAGAUUAAGGCUUGCUGGUUAUCAAUUUGAACUUUAGUCAGUCUUUUUCUCUCUCCUAUCGGU 3′<br>5′ UCAGUCUUUUUCUCUCUCCUA 3′ | 100.00 |
| **aga-mir-210** | 5′ CAUUGCAGCUGCUGACCACUGCACAAGAUUAGAAUAUGACUCUUGUGCGUGUGACAACGGCUAUUGUGGG 3′<br>5′ UUGUGCGUGUGACAACGGCUA 3′ | 100.00 |
| **aga-mir-92b** | 5′ GGGCUCCGGAUGUAGGGCGUGACUUGUGCAUAAUUUGCUGAUUUCCAAUGUCAAAUUGCACUUGUCCCGGCCUGCAGC 3′<br>5′ AAUUGCACUUGUCCCGGCCUGC 3′ | 100.00 |
| **aga-mir-190** | 5′ UUUCGGUAAGAUAUGUUUGAUAUUCUUGGUUGUUAAAUUGUUCAAUUAUCACCCAGGAAUCAAACAUAUUAUUACUGUGAC 3′<br>5′ AGAUAUGUUUGAUAUUCUUGGUUG 3′ | 100.00 |
| **aga-mir-184** | 5′ GGUGCACUCGAACCCUUAUCAUUCUUUCGCCCCGUGUGCAUUGCGAACCGACUGGACGGAGAACUGAUAAGGGCCCGGGUCACC 3′<br>5′ UGGACGGAGAACUGAUAAGGG 3′ | 100.00 |
| **aga-bantam** | 5′ AAAUGUAAUCACAGAACCGGUUUUCAUUUUCGAUCUGACUUAUUCAUUUUACAACGAGUGAGAUCACUUUGAAAGCUGAUUUUGUACAGUUAACUCAACG 3′<br>5′ UGAGAUCACUUUGAAAGCUGAUU 3′ | 100.00 |
| **aga-mir-263** | 5′ CCCUGGUACAUGUAAUGGCACUGGAAGAAUUCACGGGAUUUGUUUCAAUACUCCCGUGUUCUCUUAGUGGCAUACCCAGUACAGGG 3′<br>5′ UGUAAUGGCACUGGAAGAAUUCAC 3′ | 100.00 |
| **aga-mir-277** | 5′ GUUUUGGGGUACGUGUCAGAAGUGCAUUUACAUCGGCAUUCCGCAGUUUGAGGUAUUUGUAAAUGCACUAUCUGGUACGACAUUCCAGAAU 3′<br>5′ UAAAUGCACUAUCUGGUACGACA 3′ | 100.00 |
| **aga-mir-124** | 5′ CGUUUUUCUCCUGGUGUUCACUGUAGGCCUGUAUGUUACCUGAUUCCAUAAGGCACGCGGUGAAUGCCAAGAGCGAACG 3′<br>5′ UAAGGCACGCGGUGAAUGCCAAG 3′ | 100.00 |
| **aga-mir-10** | 5′ UUAUGUUCUACAUCUACCCUGUAGAUCCGAAUUUGUUUGAAAUUUAACAAGCGACAAAUUCGGUUCUAGAGAGGUUUGUGUGG 3′<br>5′ ACCCUGUAGAUCCGAAUUUGU 3′ | 100.00 |
| **aga-mir-13b** | 5′ UCGUGGUCGGGUCGUAAAAAUGGUUGUGCUGUGUCGAUACUUACGAAAGUUCAUAUCACAGCCAUUUUGACGAGUU 3′<br>5′ UAUCACAGCCAUUUUGACGAGU 3′ | 100.00 |

Continued on Next Page. . .

| miRNA | Alignment | Identity |
|---|---|---|
| **aga-mir-988** | 5′ CCGGUGUGUGCUUUGUGACAAUGAGAUUUUCAGUUGAAGUUCAUCCCCUUGUUGCAAACCUCACGCUGG 3′<br>5′ CCCCUUGUUGCAAACCUCACGC 3′ | 100.00 |
| **aga-mir-276** | 5′ GGUGAUUGCCAUCAGCGAGGUAUAGAGUUCCUACGUUGUUCAUAUGAAAUCUGUAGGAACUUCAUACCGUGCUCUUGGAUAGCC 3′<br>5′ UAGGAACUUCAUACCGUGCUCU 3′ | 100.00 |
| **aga-mir-219** | 5′ UUUCUAGCUCUGAUUGUCCAAACGCAAUUCUUGUUGAUACCAUUAGCUACUCAAGAGUUGUGACUGGACAUCCGUGGCUCG 3′<br>5′ UGAUUGUCCAAACGCAAUUCUUG 3′ | 100.00 |
| **aga-mir-282** | 5′ CUAAUCUAGCCUCUCCUAGGCUUUGUCUGUAAAUGGUUUCACAAUCCAGACAUAGCCUGACAGAGGUUAGGUGAAAUCUG 3′<br>5′ AAUCUAGCCUCUUCUAGGCUUUGUCUGU 3′ | 96.43 |
| **aga-mir-9b** | 5′ CACUUAUUGGGUCUUUGGUGAUUUUAGCUGUAUGUUAUUUCUUUCACAUAUAGCUUUAUCACCAAAAACCUAAUGUGUG 3′<br>5′ ACUUUGGUGAUUUUAGCUGUAUG 3′ | 95.65 |
| **aga-mir-1890** | 5′ CAGAGCUAAUUGGAGCAUUUCUUGAAGAUAUAUUUUCUGCAAACUCAUGAAAUCUUUGAUUAGGUCUGGUU 3′<br>5′ UGAAAUCUUUGAUUAGGUCU 3′ | 100.00 |
| **aga-mir-317** | 5′ CUCUGCCGCUGGGAUACACCUUGUGCUCGCUUUGCAAUUGAAAUACUCAAGUGAACACAUCUGGUGGUAUCUCAGUGGCCGGG 3′<br>5′ UGAACACAUCUGGUGGUAUCUCAGU 3′ | 100.00 |
| **aga-mir-275** | 5′ CGCGCUAAGCAGGAACCGGGACUUGAUCCAUUUUGCAAACAGUCAGGUACCUGAAGUAGCGCGCGUU 3′<br>5′ UCAGGUACCUGAAGUAGCGCGCG 3′ | 100.00 |
| **aga-mir-87** | 5′ GAUUGCUCCCGGCCCAGCCUGAAAUUUGCUAAACCUGCUGCAUAUGAGGAAAAGGUGAGCAAAUAUUCAGGUGUGUCGAAGAGUGGUC 3′<br>5′ GGUGAGCAAAUAUUCAGGUGU 3′ | 100.00 |
| **aga-mir-308** | 5′ UGUUUCGCAGUAUAUUCUUGUGAGUUUGCUUCCUUUUUAUGGGCCAAAUCACAGGAGUAUACUGUGAGAUG 3′<br>5′ AAUCACAGGAGUAUACUGUGAG 3′ | 100.00 |
| **aga-mir-279** | 5′ AAUGGGUGUGAAUCUAGUGCUUCACAUGUUUUUGCUACUGUGACUAGAUCCACACUCAUUAA 3′<br>5′ UGACUAGAUCCACACUCAUUAA 3′ | 100.00 |
| **aga-mir-92a** | 5′ UCGGCUGGAUCAAGGGCAAAAUUGUGUUUUUGAUACCAAUAUUGCACUUGUCCCGGCCUAU 3′<br>5′ UAUUGCACUUGUCCCGGCCUAU 3′ | 100.00 |

## References

1. States DJ, Gish W, Altschul SF: **Improved sensitivity of nucleic acid database searches using application-specific scoring matrices**. *Methods* 1991, **3**:66–70.
